# Emerging good practices for Translatability Assessment (TA) of Patient-Reported Outcome (PRO) measures

**DOI:** 10.1186/s41687-018-0035-8

**Published:** 2018-02-21

**Authors:** Catherine Acquadro, Donald L. Patrick, Sonya Eremenco, Mona L. Martin, Dagmara Kuliś, Helena Correia, Katrin Conway, on behalf of the International Society for Quality of Life Research (ISOQOL) Translation and Cultural Adaptation Special Interest Group (TCA-SIG), Thomas Abell, Catherine Acquadro, Ashutosh Aggarwal, Khaled Ahmed, Fatima Al Sayah, Caroline Anfray, Benjamin Arnold, Juan Ignacio Arraras, Monica Avila, Guannan Bai, Wenjing Bai, Carla Bann, Maria Barbosa, Chris Barker, Diana Barger, Susan Bartlett, Temitope Bello, Pamela Berry, Victoria Blinder, Steven Blum, Clare Bradley, Barbara Brandt, Joan Branin, Virginia Brasil, Juliana Bredemeier, Nana Brochmann, Monika Bullinger, Claire Burbridge, Dwana Bush, Elizabeth Bush, Adam Butler, Diana Camargo Lemos, Tarik Catic, Luis Cavalheiro, Eric Chan, Chih-Hung Chang, Victor Chang, Ligia Chavez, Harpreet Chhina, Wei-Chu Chie, David Churchman, Katrin Conway, Adina Coroiu, Helena Correia, Alma Cortez, Claudia Crilly Bellucci, Krister Cromm, Luz Dary Upegui, Amy DeLozier, Neha Dewan, Haryana Dhillon, Maribel Doro, Xavier Dubussche, Carolyn Eberle, Corinne Eldridge, Hannah Elwick, Sonya Eremenco, Lars Eriksson, Hans-Josef Erli, Antonio Escobar, Pedro Ferreira, Montserrat Ferrer, Thomas Finger, Marcelo Fleck, Duska Franic, Dan Froggatt, Barbara Gandek, Joshua Gandi, Olatz Garin, Cynthia Girman, Christelle Giroudet, Ari Gnanasakthy, Sabine Goldhahn, Rui Goncalves, Ana Gonzalez-Celis, Alison Greene, Alyson Grove, Claudia Haberland, Anne-Catherine Haller, Clayon Hamilton, Asha Hareendran, Melanie Hawkins, Kirstie Haywood, Carlos Hidalgo Rasmussen, Tzu-Hua Ho, Michelle Holm, Zheng-Kun Hou, Man Hung, Georgina Jones, Gloria Juarez, Valeska Kantzer, Anny Karinna Menezes, Angelos Kassianos, Manraj Kaur, San Keller, Robert Klaassen, Kunihiko Kobayashi, Shulamith Kreitler, Dagmara Kuliś, Poorna Kushalnagar, Cindy Lam, Jeanne Landgraf, Kathryn Lasch, Adelina Lear, Richard LeBlanc, William Lenderking, Lauren Lent, Gregor Liegl, Feng-bin Liu, Gail Low, Vladimir Luchkevich, Joy MacDermid, Samantha Machart, Marcos Marti-Pastor, Mona Martin, Allison Martin Nguyen, Kedar Mate, Hiroko Matsumoto, Shawn McKown, Tito Mendoza, Talia Miller, Tempei Miyaji, Kikuko Miyazaki, Lidwine Mokkink, Holger Muehlan, Wilhelm Muehlhausen, Doris Mwesigire, Eve Namisango, Michelle Naughton, Richeal Ni Riordain, Koichi Nishimura, Sandra Nolte, Rod O’Connor, Ayako Okada, Lizete Oliveira, Majid Omidikhankahdani, Ana-Maria Orbai, Richard Osborne, Ay-Woan Pan, Susan Parsons, Donald Patrick, Ranjan Pattnaik, Sylvia Paz, Sheryl Pease, Colleen Pedersen, Claudia Pereira, Marco Pereira, Timothy Poepsel, Ana Popielnicki, Fredrick Purba, Hazem Qannam, Hein Raat, Antoine Regnault, Jussi Repo, Karin Ribi, Lena Ring, Ana Maria Rodriguez, Martha Rodriguez, Martin Romero, Leo Roorda, Lara Russell, Sam Salek, Dale Sanders, Jane Scott, Kojiro Shimozuma, Sietske Sikkes, Suzanne Skevington, Adam Smith, Jeffrey Solomon, Karen Sousa, Mirjam Sprangers, Angela Stroupe, Yoshimi Suzukamo, Denise Tate, Caroline Terwee, Melissa Tinsley, Ljiljana Trajanovic, Sergiy Tyupa, Alfonso Urzua, Claudia Velez, Sheri Volger, Chonghua Wan, Hongmei Wang, Xin Shelley Wang, John Ware, Dana Weiss, Lena Wettergren, Diane Wild, Erin Wilhelm, Reg Woodleigh, Albert Wu, Frances Yang, Elizabeth Yohe Moore, Jie You, Nancy Young, Changhe Yu, Hongmei Yu, Emre Yucel

**Affiliations:** 1 Mapi Research Trust, 27 rue de la Villette, 69003 Lyon, France; 2 University of Washington, Seattle, WA USA; 3 Critical Path Institute, Tucson, AZ USA; 4 Health Research Associates, Mountlake Terrace, WA USA; 5 EORTC, Brussels, Belgium; 6 Northwestern University Feinberg School of Medicine, Chicago, IL USA

**Keywords:** Clinical outcomes assessments, Patient-reported outcomes, Translation, Cultural adaptation, Linguistic validation, Instrument development, Translatability assessment

## Abstract

This paper presents emerging Good Practices for Translatability Assessment (TA) of Patient-Reported Outcome (PRO) Measures. The ISOQOL Translation and Cultural Adaptation Special Interest Group (TCA-SIG) undertook the review of several TA approaches, with the collaboration of organizations who are involved in conducting TA, and members of the TCA-SIG. The effort led to agreement by the writing group on Good Practices for 1) the terminology to be used in referring to translatability process, 2) the best definition of TA, 3) the methodology that is recommended at each step of the process, 4) the persons involved in TA, 5) the timing of assessment, 6) the review criteria for TA, and 7) the recommendations to be made at the end of the TA process. With input from the TCA-SIG membership and in consultation with experts in the field, these emerging good practices can guide the future use of TA in the development of PROs.

**Electronic supplementary material:**

The online version of this article (10.1186/s41687-018-0035-8) contains supplementary material, which is available to authorized users.

## Background

The globalization of clinical research [1] presents ethical, scientific [2] and / or regulatory [3] challenges. One challenge is the need for patient-reported outcome (PRO) measures to be appropriate for use in different cultures to support the evaluation of treatment benefit or to detect and quantify the side-effects of treatments, under the assumption that endpoints based on these assessments collect equivalent data that can be pooled across languages. Professional societies have responded to these issues. The International Society for Pharmacoeconomics and Outcomes Research (ISPOR) Task Force for Translation and Cultural Adaptation published two papers to support the use of PRO measures in multinational contexts [4, 5]. In addition, regulators and technology assessors have focused their interest on the ability of the translated measures to express and investigate equivalent concepts across languages, as most PRO measures for global use are developed in one language and subsequently translated for use in other countries and cultures (i.e., sequential approach) [6]. In its Guidance for the use of PRO measures in drug development [7], the United States Food and Drug Administration (FDA) recommended that *“…sponsors provide evidence that the content validity and other measurement properties are adequately similar between all [translated] versions used in the clinical trial,”* further stating that the FDA would *“review the process used to translate and culturally adapt the instrument for populations that will use them in the trial.”* However, no criteria for evaluating the translation and cultural adaptation process were recommended in the FDA PRO Guidance. A review [8] suggested that translations using a rigorous and multi-step process with centralized review procedures, referred to as “linguistic validation,” may lead to translations meeting the regulators’ requirements of similar content validity. This process, however, often reveals difficulties with the source instrument when adapting the format, instructions, concepts, idiomatic expressions, response scales, or demographic items for use in different languages. To overcome these difficulties, another publication [9] suggested performance of a translatability assessment (TA) during the PRO instrument development phase before it is used in research studies or undergoes translation and cultural adaptation.

TA has been defined as *“the evaluation of the extent to which a measure can be meaningfully translated into another language”* [9]. And, as such, TA is not equivalent to a translation. The actual successful translation and cultural adaptation of an instrument into a new context can only be proven empirically, once the translation process has been finalized. A “meaningful translation” in the context of international clinical trials is one that is conceptually equivalent to the source text and culturally and linguistically appropriate in the target country to facilitate the pooling and comparison of data. TA is performed on a pre-final version of the instrument, which may be revised on the basis of the results of this review. TA is a technique that is increasingly applied on PRO measures for use either in clinical trials, or research or clinical care. Publications about the use of TA in the PRO measurement field are still scarce. A PubMed search using “translatability,” “assessment,” “review,” or “assurance” as key words retrieved only eight papers [10–17]. In seven of them [11–17], it is assumed that TA offers original and qualitative knowledge on the cross-cultural relevance of concepts, a type of information not provided by psychometric results. Only one paper out of the eight retrieved (Conway et al. [10]) has documented its value and how it is performed. The purpose of this particular study was to evaluate the extent to which a *retrospectively* conducted TA could identify the items previously singled out during the validation study as having poor content validity or poor measurement performance. The Weight module of the Youth Quality-of-Life Instrument (YQOL-W) [18, 19] was used for this appraisal of translatability. TA results confirmed problematic issues in 82% of the items which were subsequently dropped during the content validity and psychometric evaluations of the YQOL-W. However, the scarcity of official publications about TA should not overshadow the fact that in the 1990s, some researchers, for example from the International Quality of Life Assessment (IQOLA) project, the European Organisation for Research and Treatment of Cancer (EORTC) Quality of Life Group, and the World Health Organization Quality of Life (WHOQOL) Group, were also concerned by the issue of translatability assessment [20], while providing detailed protocols on how to translate and culturally adapt the original questions into new cultural contexts [21–25]. For instance, the items of the Short Form-8 (SF-8™) Health Survey were partly chosen based on ratings and comments by the translation teams [26].

A more substantial body of literature on the topic can be found in the broader field of social sciences in publications by Harkness and her team [27–29], notably the description of a similar technique called *advance translation*. In this process, experienced survey translators and survey researchers are asked to translate a pre-final version of the source questionnaire, and to comment on the problems encountered stemming either from the original questionnaire or related to the language into which it was translated. The purpose is “*to get input from different cultural and linguistic backgrounds* before *finalizing the source questionnaire for cross-cultural implementation* [29].” *Advance translation* is thus used as a method for improving the original version and the final translation process before it actually starts. As Janet Harkness writes: *“When source instruments can still be changed, translators can report back and thus help improve the source questionnaire* [28-p. 46].” In the health outcomes field, researchers are left to wonder about the current approaches to TA, what distinguishes them, and the practical recommendations that could be made to optimize TA and avoid translation difficulties during PRO instrument development and implementation.

In order to bridge this gap, the ISOQOL Translation and Cultural Adaptation Special Interest Group (TCA-SIG) has developed this emerging good practices paper for conducting TA of PRO measures. Organizations that are currently involved in conducting TA (many of whom are members of the TCA-SIG) have collaborated to arrive at an agreement for emerging good practices for the conduct and documentation of TA.

## Methods

### Paper authorship

The authorship was determined on a voluntary basis and during a discussion between the people attending the Budapest ISOQOL meeting (see Acknowledgments). Availability and experience in the TA process were also key in identifying the writing team. The authors and reviewers included a mix of business, non-profit, and academic organizations to provide a more holistic viewpoint.

### Identification of organizations performing TA of PRO measures

In May 2013, a Google internet search was performed to identify translation companies clearly indicating TA in their services. The search retrieved eight names (i.e., Mapi, cApStAn, Sergius Linguistic Guidance, Round Peg Research, PharmaQuest, Validata, TransPerfect, and Corporate Translations). In addition, the TCA-SIG membership was consulted, and five other organizations which perform TA were identified, i.e., the Department of Medical Social Sciences at Northwestern University, Evidera, FACITtrans, ICON, and Health Research Associates. Letters were sent to invite each organization to be part of the agreement process. Eight organizations, out of 13 contacted, agreed to participate in sharing their current TA practices (2013), i.e., Corporate Translations, the Department of Medical Social Sciences at Northwestern University, Evidera, FACITtrans, ICON, Health Research Associates, Mapi, and PharmaQuest.

### Information collection

Each organization was asked to answer a set of questions compiling information on its methodological approach to TA as a first step to identify common practices for building an agreement. In order to facilitate the review, analysis, and comparison of approaches, the questions were organized according to the following categories: (1) terminology used to refer to the process, (2) definition of TA, (3) steps of the process, (4) people involved in the process, (5) timing of assessment, (6) review criteria, and (7) recommendations.

### Agreement and review process

During the analysis and comparison of approaches, commonalities and disparities between methodologies as well as original perspectives were highlighted. On the basis of the analysis and comparison efforts, the core team (i.e., writing team) defined their agreement for each of the categories, i.e., 1) terminology, 2) definition, 3) steps, 4) people, 5) timing of assessment, 6) review criteria and 7) recommendations. The core team held monthly teleconferences (over a period of 12 months) to discuss issues that arose during manuscript development and to produce a first draft paper. The tables summarizing the approach of each organization (category by category) and the details of the analysis of differences and commonalities can be found in the supplementary files (Additional file 1: Table S1 to S7).

The first draft manuscript was submitted for review and comments to a primary review team which included members of the organizations not involved in the writing process and researchers in PRO development and cross-cultural issues (see acknowledgements). Comments were discussed and incorporated. A second draft was presented to all members of the TCA-SIG for review. All comments from the TCA-SIG membership review were addressed. The revised manuscript was submitted to the ISOQOL Board and ISOQOL membership for review and approval. All comments from the ISOQOL membership were dealt with and changes were made to the manuscript to develop the final version.

## Results

### Terminology

With only very slight variation, all organizations refer to the process as translatability assessment (Additional file 1: Table S1).

To promote a single formulation and based on our review, the working group proposed the term “translatability assessment” to refer to the evaluation of the translatability of a PRO measure into other languages.

### Definition

Based on the review and analysis of all definitions (Additional file 1: Table S2), the following elements and considerations were retained as important for inclusion in the agreed definition:

- The definition of TA should apply to any source language.
- The definition should remain short but clarify the goal, means, and expected results. In addition, while some organizations suggested modifications to the source text, none recommended the possibility of providing alternative choices of wordings on which future translations should be based, when modifications of the original are not needed or accepted by the developer. This point should be added in the definition.
- The definition should provide reference to the context in which the TA is used (e.g., global studies, international clinical trials).
- It should refer to “translation” without specifying the number of languages or language families. Because every project requiring TA has its unique differences, the decision about how many languages and what languages to include needs to be tailored to reasonably support the needs of the specific project. Considerations should include whatever is known about the target use of the measure, and the diversity of languages and language families to potentially be included in the use of the measure. It may not be necessary to sample every language on the list planned for translation, especially those having similar structural patterns and grammar rules. However, consideration must be given to the languages that present extreme differences and particular problems in order to have an effective range of translatability assessed and meaningful information provided during the development of a measure.
- It should indicate the timing of the translatability assessment.
- The use of “text” instead of “item wording” is preferred, since TA is performed on all the components of a PRO measure (title, instructions, items, and response categories).
- The definition should not refer to the participation of the developer, which should be referred to in the category “people involved.” The idea is to favor a short definition (or at least as concise as possible), and explain later (in the category of people involved) and in detail the reasons why the involvement of the developer in the TA process is desirable.

Recommended definition:

Translatability assessment (TA) of a patient-reported outcome (PRO) measure is the review of its source text preferably during the development stage, prior to its use, in order to determine its suitability for future translations in multilingual studies. In this context, the translation process aims to create conceptual equivalence to the original in a way that allows data from multiple languages to be compared. The goal of TA is to facilitate future translations and use of the measure in global studies by: 1) identifying and categorizing potential translation issues in the source text and 2) providing alternative choices of wordings on which translations can be based and/or recommendations of how to modify the source text so that future translations are conceptually and culturally appropriate for the target populations.

### Steps

Based on the review and analysis of the steps proposed by all organizations (Additional file 1: Table S3), we recommend the following steps for translatability assessment:

- Four major steps should be considered: preparation, review, recommendations, and writing of a report.
- Each step and sub-step should be thoroughly described.
- The target population and any special requirements need to be considered in the review process. For instance, reviewers need to consider language that is appropriate depending on the cognitive abilities of the target population [e.g., development (in children) or decline (in the elderly)].
- The preparation step should include:
  - ◦ The itemization of each element of source text that presents a concept.
  - ◦ A concept definition in order to clarify the meaning of the source text: a clear, consistent, and relevant definition of concepts will provide a higher chance that the reviewers would understand the source text as intended by the developer.
  - ◦ The development of a table for reporting each TA review.
  - ◦ The development of an overview of the qualifications of the reviewer(s) involved.
  - ◦ The provision of clear and detailed instructions to the reviewers.
- The review step should include:
  - ◦ The analysis of the translatability of each part of the source text (i.e., title, instructions, items, and response options) according to specific review criteria (described below) to identify problematic issues.
  - ◦ Ratings to describe the difficulty of translating the wording of the source document.
    - ◦ **Level 1 – No Difficulty**: No problems expected in developing a rendering that faithfully captures a concept that is equivalent to the source text.
    - ◦ **Level 2 – Minor Difficulty**: Minor departure in language would be necessary to maintain conceptual equivalence with the source wording. *Examples include* changes in word or phrase order, substitute phrasing, use of the closest available verbal tense, etc. A conceptually equivalent word or phrase does exist in the target language, but for reasons of grammatical or linguistic fluency, it must be expressed in an alternative way or with a distinct syntactical structure in the target language.
    - **Level 3 – Major Difficulty**: A significant departure from the source language would be necessary to render the item into the target language. *Examples include* having no corresponding term or phrase with the same meaning (or scope of meaning) as the original source text, or making an addition or omission necessary in order to convey the same concept as the source text. For example, an available idiom or more than one term might be necessary to match the scope of meaning of a source item. Also, the item may contain words that would typically be translated with a term already used elsewhere in the questionnaire (which have distinct meanings in the source language but are expressed with a single term in the target language).
    - **Level 4 – Extreme Difficulty**: Translators would face extreme difficulty to appropriately convey the concept presented in the source text with clarity. There may be cultural taboos or other sensitivity issues that would make effective rendering of the concept into a given language not possible. Specific terminology may not exist for that concept or the concept may be completely inapplicable to the target culture. This level of difficulty might be grounds to recommend 1) either that the underlying concept be reconsidered and expressed in a completely different way that is more cross-culturally appropriate, 2) or, if revision is impossible, recommend that the concept be dropped from the measure because of the high likelihood of differential item functioning that will create problems with pooling the data for future analysis.

One or more reviewers assess the difficulty of each component of the item using this scale, and in the case of multiple reviewers, all ratings are averaged across reviewers to generate the difficulty rating. Ratings of 3 or 4 are likely to be associated with recommendations for revisions to the source wording to address the threat to conceptual equivalence, while a rating of 2 may result in suggested alternative wording for future translations that is considered conceptually equivalent but no proposed changes to the source text. These ratings are intended to be used as a tool to communicate the potential threats to conceptual equivalence to the developer in conjunction with detailed explanations of the problem and proposed solution.

The inclusion of a readability assessment (grade or reading level) in the review step raised a lot of discussion and comments from TCA-SIG members, i.e., how to do it, who should do it, etc. The idea was to identify possible issues in the register of the language used in the source text. We decided to recommend that this step should be completed by the developer of the original measure, acknowledging the fact that TA might detect, in the original measure, wordings or syntax that may be too complicated or difficult to understand by lay people and, therefore, might lead to translatability issues.

- The recommendation step should include:
  - A detailed description of the advice proposed to address all problematic issues.
  - A consultation with the developer of the source text to review all recommendations and decide upon any change to be made to the source text.
- The last step involves the completion of a final report outlining all processes undertaken, findings, and recommendations.

### People involved

Following suggestions by all organizations (Additional file 1: Table S4), we recommend that the following people be involved in the process:

- The developer(s) of the PRO measure under review;
- Reviewer(s) who will perform the TA;
- A project manager.

Table 1 below illustrates the categories of people involved in the TA process, as well as their qualifications and rationale for their involvement.

**Table 1:** People involved in the Translatability Assessment (TA) process

People to be involved	Qualifications	Rationale
Developer(s) of the patient-reported outcome (PRO) measure	Researcher(s) involved in the development of the instrument being assessed.	Developer(s) know(s) the purpose and intent of the measure and of each item. Best qualified to explain the concepts behind each item and the choice of response categories. Developer(s) make(s) all final decisions in response to the TA recommendations.
Reviewer(s)	Knowledge of language(s) other than the source language, at a near-native level. Expert(s) in linguistic competency (i.e., able to deconstruct and analyze a sentence, understand the meaning of each component of a sentence and how they interact). Experienced in multilingual projects of translation of PRO measures. A minimum of 5 years of continuous experience translating PROs is recommended.	Each reviewer is empowered to provide recommendations on the suitability of the source text for translation and to suggest modifications to the source text to improve future translatability.
Project Manager	Experienced in linguistic validation and in conceptual analysis of PRO measures.	The contribution of a project manager is needed and valuable for projects involving many target languages (when they are identified). He/she is the key person who coordinates the process, facilitates the communication between the reviewers and the developer of the measure, and consolidates all reviewers’ comments. He/she provides support to the developer during the development of the concept definition.

Gender issue among TA reviewers should be addressed as research has shown that language and gender may be intertwined [30]. We recommend using reviewers from both genders when possible in order to prevent any bias.

We did not specify a definite number of reviewers as the decision of how many people to involve is often project-specific and is defined case-by-case. Cost may also be a criterion to be taken into consideration. The review of all methodologies (see S4) shows that the number of people to be involved varies greatly, especially the number of reviewers/translators involved (from 1 to 15). Along these lines, we did not specify a number of language families to be represented as this is decided case-by-case as well.

As for the initiation of the TA process, two situations should be considered:

- In the case of the development of a new measure, the initiator is the developer whether he/she is from academia or industry.
- In the case of an existing measure, TA can be initiated by whoever needs it. However, if the TA leads to suggest changes to the original measure, then the author of the original should be made aware of it.

### Timing of assessment

Based on our review of the timing of TA proposed by all organizations (Additional file 1: Table S5), we advise that:

- TA should be performed as early as possible during the development of the PRO measure, when adjusting the conceptual framework and drafting the instrument, preferably before quantitative testing with patients. It is optimal during the qualitative phase of development, before finalization of the wording, while changes can still be made, and before use in quantitative research. It is less useful to conduct TA after the psychometric evaluation has been performed because changes to the content and wording at that point may change the measurement properties of the measure. However, it may still be beneficial to conduct TA on a measure after psychometric evaluation in order to prepare for translation and generate alternative wording for any items that are identified as problematic by TA but cannot be changed in the measure. In this case, TA will clearly point out the translation problems with existing measures. The disadvantage to conducting TA late in the lifecycle of the measure is that fewer options are available to address issues detected by TA, so we consider the good practice to perform TA as early as possible during instrument development.
- TA should be iterative when new wording is generated. If cognitive interviews with patients lead to changes in the wording during the qualitative phase of development, a TA should ideally be performed again on the final text. The number of iterations should follow the number of changes made to the wording. It is of foremost importance that the translatability of all new wordings be assessed, especially prior to the final round of cognitive interviews during which the measure content is confirmed.

**Fig. 1:**
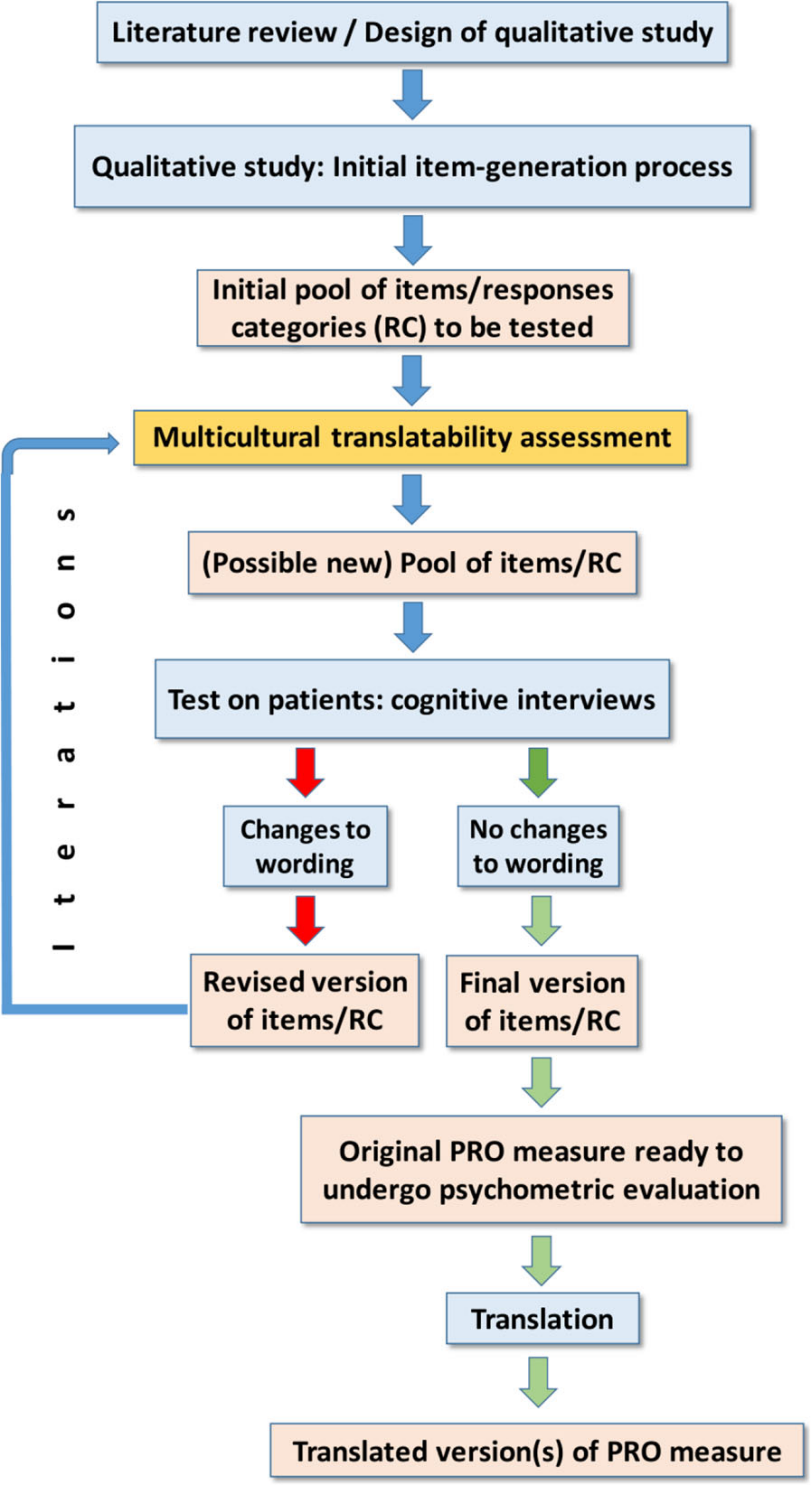
Timing of TA during PRO measure development

Figure 1 below illustrates an example of timing of TA during the qualitative phase of PRO measure development.

### Review criteria

Based on the review and analysis of review criteria suggested by all organizations (Additional file 1: Table S6), we recommend the following:

- The review criteria should be divided into three categories:
  1. Culture
  2. Language
  3. Item construction

See Table 2 for a definition of each category.

**Table 2:** Categorization of translatability review criteria

Categories of translatability review criteria*		Definition
1. Culture		A word or formulation in the original is culturally loaded in the target context due to societal or cultural conventions (e.g.., eating or clothing habits, religious taboos). The usage of certain words or phrases based on the culture of a given society may be improper in the target language and/or culture. For instance, certain foods are not eaten in target countries and should be replaced in the translations. *For example, starchy foods (*e.g. *potato, bread) becomes starchy foods (*e.g. *rice, pasta, chapatti).*
2. Language		
	2a. Meaning (Semantics)	Semantics concerns meanings, which are both denotative, i.e. the literal word (lexis), and connotative, namely the set of cultural and/or subjective associations implied by a word in addition to its literal explicit meaning. This category includes lexical differences. For instance, English has a slightly larger lexicon than French. Therefore, some French words have no direct equivalent in English and would need the use of paraphrases. **Example 1**: The literal translation of the word “frustrated” in French (i.e., “frustré(e)”) has a very different connotation than the US English meaning. The word “frustré(e)” is often associated to dissatisfaction. In general, the English word “frustrated” covers several feelings (annoyed, powerless, disappointed, and angry) that are not embedded in one word in many other target languages. **Example 2**: The meaning of “depressed” can range from “feeling a little sad” to “clinically depressed” in English depending on the context. However, in some languages, only the latter meaning applies, so developer(s) might consider a more specific word instead of “depressed.” **Example 3**: “Maladie” in French is a unique word for which English has several terms, i.e., condition (general word), illness (perception, before you go to the doctor), disease (when you come from the doctor, i.e., with a medical diagnosis)
	2b. Use (Pragmatics, Idiomatic expressions)	The practicalities of how a language is used in its everyday context may be different between the source and target language. For example, one language may have more social registers than another (there are a number of different forms of addressing a person in Japanese, whereas English may only have one) and the idiosyncrasies of one language (repetitions, focus on particular words, use of particular idiomatic expressions, etc.) may not be found in another. *For instance, “I feel downhearted and blue* *” translated by an equivalent of “I feel downhearted and sad” or “I feel downhearted and depressed.”*
	2c. Syntactic (syntax, grammar and punctuation)	Grammatical and syntactical possibilities vary across languages and may impact the identification of conceptually equivalent alternatives in a target language. The structure and grammar of the source and target language diverge. **Example 1**: The use of a verbal passive form in the original may not be possible in some target languages where active form is a more natural verbal construction. **Example 2:** The placement of the recall period might differ in some target languages. In English, it often goes at the beginning or end of the item, but in other languages it might be grammatically necessary to place it in the middle of the item.
3. Item construction		
	3a. Item vague, ambiguous	The meaning of the item or words within the item are unclear and can be understood in multiple ways in the source text, leading to potential mistranslation in target languages if the wrong nuance is chosen.
	3b. Use of double negative	A double negative in an item or in conjunction with a negative response choice makes the response and its interpretation difficult because in some languages, the double negative creates a positive meaning, while in other languages, the double negative merely reinforces the negative concept.
	3c. Readability issues	The language used in the original is too high a reading level for clarity and might impair the understandability of the original and, therefore, impact the future translations. Readability assessment may be needed if not previously conducted by measure developer.
	3d. Redundancy between items	Two items may express the same concept or close concepts that would be translated the same way
	3e. Lack of coherence with concept	The terms used in the item do not seem to adequately convey the meaning of the concept to be measured.
	3 f. Lack of coherence of response scale with item	The response scale does not fit the phrasing of the item.
	3 g. Two concepts within one item	The item may express two different concepts that may confuse the respondents.

*See Brislin 12 guidelines for writing translatable English [31]

### TA recommendations

Based on the review and analysis of the TA recommendations suggested by all organizations (Additional file 1: Table S7), we propose the following recommended actions resulting from the review step of each element of the text:

- No change to the original wording. The wording of the source text is suitable for international translation and does not require any changes.
- No change to the original wording, but suggestions for alternative wording (based on concept elaboration) suitable for translation to address known issues. This recommendation is suggested when the source wording is the best way to express the concept in the source language but does not translate well.
- Change to the original wording to address issues identified by TA that can threaten the measurement of the concept in other languages.
- Consider removing wording because of extreme degree of difficulty to translate in the future. The developer may decide to keep the item to explore how the item functions during psychometric evaluation before deciding to remove it.

Table 3 summarizes the good practices for the TA of PRO measures.

**Table 3:** Summary of Translatability Assessment (TA) Good Practices for patient-reported outcome (PRO) measures

Category	Description
Terminology	Translatability Assessment (TA)
Definition	Translatability assessment of a patient-reported outcome (PRO) measure is the review of its source text preferably during the development stage, prior to its use, in order to determine its suitability for future translations in multilingual studies. In this context, the translation process aims to create conceptual equivalence to the original in a way that allows data from multiple languages to be compared. The goal of TA is to facilitate future translations and use of the measure in global studies by 1) identifying and categorizing potential translation issues in the source text, and 2) providing alternative wordings on which translations can be based and/or recommendations of how to modify the source text so that future translations are conceptually, culturally appropriate for the target populations.
Steps	Four major steps should be considered: preparation, review, recommendations, writing of a report.
People Involved	- The developer(s) of the PRO measure under review; - Reviewer(s) who will perform the TA; - A project manager.
Timing of Assessment	TA should be performed during the development of the PRO measure, as early as possible, when adjusting the conceptual framework and drafting the instrument. It should happen during the qualitative phase of development, before finalization of the wording, while changes can still be made to the wording, and before use in quantitative research. It is less useful to conduct TA after the psychometric evaluation has been performed, and if performed during this later stage, options to address issues detected are limited to providing alternative wording for future translations which may still be beneficial. - TA should be iterative when new wording is generated. If cognitive interviews with patients lead to changes in the wording during the qualitative phase of development, a TA should be performed again. The number of iterations should follow the number of changes made to the wording. It is of foremost importance that the translatability of all new wordings be assessed before the final round of cognitive interviews.
Review Criteria	The review criteria should be divided in three categories: culture, language, and item construction.
Recommendations	- No change to the original wording. - No change to the original wording, but suggestions for alternative wording suitable for translation to address known issues. - Change to the original wording. - Consider removing wording because of extreme degree of difficulty to translate in the future.

## Discussion/conclusion

With the increase of patient-centered initiatives aimed at obtaining patients’ perspectives on the impact of medical treatments in multinational clinical trials, the need for PRO measures in many languages has become of paramount importance. Although good practice recommendations for the translation and cultural adaptation process as well as reviews of methods of translation are available [4, 5, 8], no recommendations on how to assess the translatability of an original PRO measure during its developmental phase have been published. While we found only one publication that has demonstrated the value of performing TA [10], it involved a retrospective design. This publication found that TA results confirmed problematic issues in 82% of the items dropped during the content validation and psychometric evaluation of the Youth Quality-of-Life Instrument–Weight module.

The ISOQOL TCA-SIG undertook the review of several translatability assessment approaches, and, on the basis of the analysis and comparison efforts, the TCA-SIG proposed its views on which terminology should be used to refer to the process, what the best definition of TA should be, which methodology should be followed at each step of the process, who should be involved in TA, what the best review criteria are, and which recommendations should be made at the end of the TA process. A summary of the agreed good practices is presented in Table 3.

TA should be viewed as a first step in reaching conceptual equivalence between the original measure and its future translations. It does not replace the evaluation of differential item functioning or the qualitative work done either during the simultaneous development of a measure in different countries or during the translation process of a measure developed in one language and to be used in several other countries. TA could also constitute an important dimension when outcome measures are being evaluated and selected for use in research (in line with the COMET initiative [32, 33] for instance).

Most of the TA research performed to date has been carried out on measures developed in English. We have assumed that the principles of good practices that we are proposing apply to other source languages. However, we do not have sufficient empirical data to confirm this assumption.

The TCA-SIG plans to investigate ways to address the need for empirical evidence of the value of conducting TA during the PRO measure development process, whatever the source language of the original. We would like to encourage researchers to publish more evidence on the importance of performing a TA during the PRO measure development phase. Too often these steps are not reported, though performed, and the item reworded, before being issued. It would be helpful if those changes were made fully transparent in the literature. The TCA-SIG plans to collect examples of TA studies and present examples of items suggested for change and the reasons for those changes. The aim is also to follow up on the instrument development studies and illustrate how TA has enhanced the evolution of the measure. The development of a database of experience would be extremely helpful.

Most of these principles of good practice may apply to the translatability of the other types of clinical outcome assessment (COAs), i.e., clinician-reported outcome (ClinRO), observer-reported outcome (ObsRO) and performance outcome (PerfO) measures. Further research is needed to investigate whether specific amendments to the PRO measure TA good practices should be made for other COAs.

During our reviewing and writing process, we received several comments about timing of assessment from TCA-SIG reviewers, arguing that TA could be done at any time of the life cycle of a PRO measure. We agree that other uses might be envisaged, such as a retrospective evaluation of a fully developed original measure (i.e., psychometrically evaluated) for research purposes. For instance, researchers might want to know if this measure would be suitable for their country and language(s), and apply TA to this end. This might lead to the decision of not using this specific measure if TA reveals issues in the original that cannot be changed. However, we would like to reiterate that our research was primarily focused on the prospective use of TA, i.e., during the development phase of a PRO measure.

## Additional file

Additional file 1: **Table S1** Terminology . **Table S2** Definitions. **Table S3** Steps used by each organization. **Table S4** People involved. **Table S5** Timing of assessment. **Table S6** Review criteria. **Table S7** Recommendations (DOCX 56 kb)

## Abbreviations

EORTC: European Organisation for Research and Treatment of Cancer
FDA: Food and Drug Administration
IQOLA: International Quality of Life Assessment
ISOQOL: International Society for Quality of Life Research
ISPOR: International Society For Pharmacoeconomics and Outcomes Research
PRO: Patient-Reported Outcomes
SF-8: Short Form-8
TA: Translatability Assessment
TCA-SIG: Translation and Cultural Adaptation Special Interest Group
WHOQOL: World Health Organization Quality of Life
YQOL-W: Weight module of the Youth Quality-of-Life Instrument
