# Supplementary material for: Emerging good practices for Translatability Assessment (TA) of Patient-Reported Outcome (PRO) measures
Source: J Patient Rep Outcomes. 2018 Feb 21;2:8. doi: 10.1186/s41687-018-0035-8 (PMC5935017; doi:10.1186/s41687-018-0035-8)
Supplement: Supplementary file 1 — Table S1 Terminology . Table S2 Definitions. Table S3 Steps used by each organization. Table S4 People involved. Table S5 Timing of assessment. Table S6 Review criteria. Table S7 Recommendations (DOCX 56 kb) [file 41687_2018_35_MOESM1_ESM.docx]

**Supplementary Files**

**S1. Terminology**

Table 1 provides an overview of the terminology used by each organization.

**Table 1. Terminology**

| **Company / Organization (in alphabetical order)** | **Terminology used** |
| --- | --- |
| **Corporate Translations, Inc** | Translatability Assessment |
| **Department of Medical Social Sciences, Northwestern University (NU)** | Translatability Assessment; Translatability Review |
| **Evidera, Inc** | Translatability Assessment; Translatability Review |
| **FACITtrans, LLC** | Translatability Assessment; Translatability Review |
| **Health Research Associates, Inc** | Translatability Assessment |
| **ICON plc** | Translatability Assessment; Translatability Assurance  (preference for Assessment) |
| **Mapi** | Translatability Assessment |
| **PharmaQuest Ltd** | Translatability Assessment |

Analysis of commonalities and disparities/originalities

Regarding the terminology used to refer to the process, all organizations use the expression “translatability assessment.” In addition, three organizations propose “translatability review” (i.e., Evidera and FACITtrans, and NU), and one, “translatability assurance” with a preference for “translatability assessment” (i.e., ICON).

**S2. Definition**

Table 2 provides an overview of the definitions proposed by each organization.

**Table 2. Definitions**

| **Company / Organization**  **(in alphabetical order)** | **Definition** |
| --- | --- |
| **Corporate Translations, Inc** | The objective of the translatability assessment is to assess the conceptual clarity and translatability of a PRO measure in order to determine if the concepts of the source document can be adequately captured in translation. |
| **Department of Medical Social Sciences, Northwestern University (NU)** | Translatability Review consists of evaluating the text used in the source language to identify potential conceptual and linguistic issues which could render translation into other languages difficult, impair cross-cultural research, or impact the use of a particular technology to administer the measures. Reviewers may recommend deletion of certain items or offer alternative wording solutions more suitable for a culturally diverse population, for translation, and for the measure’s mode of administration. |
| **Evidera, Inc** | Translatability assessment is the evaluation of the source text of a clinical outcome assessment (COA) instrument to determine its suitability for future translation in global studies or clinical trials to enable conceptual equivalence between source and target versions and enhance the pooling of data from different translations.  The goal of TA is identify problematic text in the source instrument and suggest modifications to the source text to improve the likelihood of a suitable translation in the future, but occurs well before the translation process is undertaken. |
| **FACITtrans, LLC** | Translatability Assessment is the process of ensuring that item wording is suitable for culturally diverse populations and appropriate for harmonized translation into multiple languages. Translatability Assessment refers to the proactive effort to identify and categorize potential issues in source wording working hand in hand with the developers of COA measures. |
| **Health Research Associates, Inc** | Assessment of the suitability of language proposed in COAs in development stages for cross cultural translation and linguistic validation (looking at the understandability of the items and the ability to relay the intended concept for evaluation). |
| **ICON plc** | Translatability Assessment is the review of source text to determine whether there are likely to be any translation problems and unclear concepts and to provide recommendations to resolve them |
| **Mapi** | Evaluation of the extent to which a PRO measure can be meaningfully translated into another language.  A “meaningful translation” in the context of international clinical trials is one that is conceptually equivalent to the source text and culturally and linguistically appropriate in the target country to facilitate the comparison and pooling of data.  The goal of a TA is to identify translation difficulties and suggest items to be deleted before embarking on the translation process as such. |
| **PharmaQuest Ltd** | A translatability assessment is used to assess the suitability of a measure for translation into a wide range of languages, for use in a variety of cultures.  The aim is to ensure that it will be possible for translations of the text to be semantically accurate representations of the source text and also culturally appropriate for the target countries. This is done by adapting any elements of the text that are linguistically or culturally bound to the source language or country, and ensuring that the text contains only universally meaningful ideas that are expressed in a clear way. |

Analysis of commonalities and disparities/originalities

All organizations mention the word “translation” either in reference to the suitability of the source text [for translation] (i.e., Evidera, FACITtrans, Health Research Associates, NU and PharmaQuest) or to conceptual equivalence (i.e., Corporate Translations) or to problematic issues (i.e., ICON), or meaningfulness (i.e., Mapi).

Eight organizations use “source” as an adjective to qualify “document” (i.e., Corporate Translations), “text” (i.e., Evidera, ICON, Mapi, NU, and PharmaQuest), “instrument” (i.e., Evidera), and “wording” (i.e., FACITtrans). Only two use the term “target” to qualify country(ies) (i.e., Mapi, PharmaQuest).

Seven companies combine definition and objective, either in a very direct way [i.e., “TA is…. The objective/goal/aim is…” (Evidera, Mapi, and PharmaQuest), or more indirectly [i.e., “TA is…to determine… and to provide” (ICON) or “TA is… TA refers to the proactive effort to identify and categorize…” (FACITtrans); “Assessment of… for…” (Health Research Associates); TA consists of evaluating…to identify issues…recommend solutions (NU)]. In addition Health Research Associates mentions the means to achieve the objective: “looking at the understandability of the items and the ability to relay the intended concept for evaluation.” One company provides only the objective with mention of the means to achieve it [i.e., “…is used to… The aim is…,” “This is done by adapting…” (Corporate Translations)].

Six companies use words in relation to the concept of estimation [i.e., “to assess” (Corporate Translations, PharmaQuest); “assessment” (Health Research Associates); “evaluation/evaluating” (Evidera, Mapi, NU)].

The terms “cultural” or “culturally” are used by five organizations in relation to relevance of the translation for populations or countries [i.e., “culturally diverse populations” (FACITtrans, NU)] or target countries [i.e., “culturally appropriate in the target country” (Mapi); “culturally appropriate for the target countries” (PharmaQuest)] or to qualify the term translation [i.e., “cross-cultural translation” (Health Research Associates)].

Six organizations make reference to concepts. Two mention conceptual equivalence between source and target versions [i.e., “to enable conceptual equivalence between source and target versions” (Evidera); “is conceptually equivalent to the source text” (Mapi)]. Three elaborate around the relevance of the concepts in translations [i.e., “to determine if the concepts of the source document can be adequately captured in translation” (Corporate Translations); “ability to relay the intended concept” (Health Research Associates); “to identify potential conceptual and linguistic issues” (NU)]. And two refer to the clarity of concepts [i.e., “to assess the conceptual clarity of a PRO measure” (Corporate Translations); “to determine whether they are likely to be unclear concepts” (ICON)].

Five organizations use the words “suitable” and “suitability” in reference to translation, culture, language or measure, either in the definition or in the objective [i.e., “to determine its suitability for future translation,” “to improve the likelihood of a suitable translation in the future” (Evidera); “ensuring that item wording is suitable for culturally diverse populations” (FACITtrans); “assessment of the suitability of language” (Health Research Associates); “alternative wording solutions more suitable for a culturally diverse population, for translation, and for the measure’s mode of administration” (NU); “to assess the suitability of a measure for translation into a wide range of languages” (PharmaQuest)].

In the objective, four organizations refer to the identification of problematic issues [i.e., “identify problematic text in the source instrument” (Evidera); “to identify potential issues in source wording” (FACITtrans); “to determine whether there are likely to be any translation problems” (ICON); “to identify translation difficulties” (Mapi); “to identify potential conceptual and linguistic issues” (NU]. Only one introduces the idea of categorizing potential issues in the source wording (i.e., FACITtrans)].

Four organizations relate to future recommendations [i.e., “suggest modifications to the source text” (Evidera); “to provide recommendations to resolve them” (ICON); “to suggest items to be deleted” (Mapi); “recommend deletion of certain items or offer alternative wording solutions” (NU)].

Three organizations refer to timing of assessment [i.e., “but occurs well before the translation process is undertaken” (Evidera); “in development stages” (Health Research Associates); “before embarking on the translation process as such” (Mapi)].

Only two organizations refer to PRO measures (i.e., Corporate Translations and Mapi); and three, to COAs (i.e., Evidera, FACITtrans, Health Research Associates). One organization refers to “item wording” without specifying the origin of the items, except at the end of the definition when referring to the developers of the COA measures (i.e., FACITtrans). ICON only refers to the “source text”, NU refers to “measures”, and PharmaQuest to “a measure” without specifying if it is a PRO or a COA.

In the objective, two organizations mention pooling of data [i.e., “to enhance pooling of data” (Evidera); “to facilitate pooling of data” (Mapi)].

Two organizations provide some notions of context [i.e., “in global studies or clinical trials” (Evidera); “in the context of international clinical trials” (Mapi)].

Two organizations refer to meaningfulness, however in two different contexts. In the definition, Mapi introduces the notion of “meaningful translation,” while, in the objective and means to attain the objective, PharmaQuest mentions the notion of “ensuring that the text contains only universally meaningful ideas that are expressed in a clear way.”

Only three companies cite multiplicity of languages in reference to translation [i.e., “for harmonized translation into multiple languages” (FACITtrans); “translation into other languages” (NU); “for translation into a wide range of languages” (PharmaQuest)]. And only FACITtrans and NU refer to the “suitability for culturally diverse populations.”

When referring to the appropriateness of the future translations, the term “linguistically” is used by Mapi (i.e., “linguistically appropriate in the target country”), and the term “semantically” is used by PharmaQuest (i.e., “to be semantically accurate representations of the source text”).

One organization includes a reference to people involved [i.e., “working hand in hand with the developers” (FACITtrans)].

FACITtrans is the only one to refer to “harmonized translation.”

NU is the only one to refer to translatability issues that can “impact the use of a particular technology to administer the measures.”

**S3. Steps**

Table 3 provides an overview of the steps proposed by each organization.

**Table 3. Steps used by each organization**

| **Company / Organization**  **(in alphabetical order)** | **Steps** |
| --- | --- |
| **Corporate Translations, Inc** | - Identification of concepts, conducted with assistance from the instrument developer if possible. - Review of original PRO text by Survey Research Analyst, seeking to identify any concepts or terms that may present issues during future translation activities - Review of original PRO text by native-speaking professional translators experienced in PRO translation and validation. Ideally, representatives from each of the following language groups should be included: Western European, Eastern European, Indian, Scandinavian, Asian and Middle Eastern. - Review, analysis and consolidation of all feedback by Survey Research Analyst and Project Manager - Completion of final report outlining all processes undertaken, findings, and recommendations. |
| **Department of Medical Social Sciences, Northwestern University (NU)** | Review comments are included in the source document or compiled in one table next to each item or piece of text being evaluated.  Reviewer(s) use criteria list and guidelines provided and start each comment with a key word/expression (e.g., “idiomatic”, “redundant”, “ambiguous”, etc.) to facilitate post-review final recommendations and decisions.  Reviewer(s) provide explanation of the issue and, if possible, a suggestion for alternative wording where applicable.  Reviewer(s) highlight items or text where changes are needed or highly recommended in order to improve clarity and translatability.  When assessement is done by multiple reviewers, the lead reviewer makes a final recommendation based on all input. |
| **Evidera, Inc** | 1. Development of the TA table used to conduct the assessment. 2. Review of the original wording and identification of problems and issues. 3. Lexibility assessment (grade or reading level) to ensure that the reading level is appropriate for the target audience. 4. Rating on a 0 to 5 scale regarding the difficulty to translate the current wording:   Difficulty Rating  0 = no difficulty to translate  1 = minor difficulty to translate, revision offered  2 = moderate difficulty, change suggested  3 = a lot of difficulty, change strongly recommended  4=a great deal of difficulty, change strongly recommended  5=impossible to translate or find equivalent meaning, consider removing from source   1. Recommendation to address the difficulty, issue noted, or reading level concern on a per item basis. 2. Production of report on the findings |
| **FACITtrans, LLC** | 1. Items are provided by developer for review  2. Items are reviewed by professionals with training in the area and categorized according to analysis criteria below.  3. If multiple reviewers are involved then reviewer feedback must be synthesized and reconciled.  4. Final recommendations are made on an item by item basis in the form of a report. |
| **Health Research Associates, Inc** | 1. Developers identify intended meaning of concepts for current draft text 2. Project manager sets up translatability instructions, tables, selects language consultants 3. Between 4 and 6 “representative” languages selected (based on project specific criteria) 4. Relative difficulty levels assigned to all text by language consultants, with examples and suggestions provided 5. Reviews collated by project manager and presented to COA development scientists to discuss and combine with final cognitive assessment work going on with the developing measure 6. Decisions made and tested while cognitive interviews are still ongoing 7. Final decisions and implemented changes documented and report developed. |
| **ICON plc** | Review by senior project managers (2), review by linguists representing different groups (5-15) (e.g., languages using Chinese characters, Indian languages, Cyrillic languages, Romance and Germanic languages, Latin American Spanish, African languages (Zulu etc.). The linguists are provided with a grid and asked to look for translation and cultural difficulties and come up with potential solutions, the PMs compile results from different countries in a grid and then hold a translatability meeting, if there are significant issues from a particular language we will negotiate with the linguist, otherwise all results and recommendations will be returned to the developer |
| **Mapi** | - Identification of concepts underlying the instructions, items, and response choices to answer two essential questions: “what is being investigated?” (i.e., what is the concept) and “how is this expressed and what value do you place on it?” (i.e., what is the construct); - Review of the original wording. Issues encountered during the process are divided in two categories, i.e., cross-cultural and structural; - Production of recommendations; - Production of a report on the findings. |
| **PharmaQuest Ltd** | 1- Concept elaboration. A detailed description is written for all elements of the text, to explain the intended meaning and function of each item and to clarify any ambiguous terms. This is reviewed by the instrument developer, who responds to any queries raised, and then is made available to the whole project team for reference.  2- International review. A review is carried out by a minimum of 5 translators, but usually 10, whose native languages are chosen to represent as wide a range of language groups and cultures as possible. Each translator reviews the text and, for each item, assesses the extent to which a more or less direct translation would be both linguistically possible in the target language and culturally appropriate in the target country. They are requested to advise on any items that would require restructuring or rephrasing, and to comment on any other aspects of the text that may not translate well, either linguistically or culturally, in their language and country.  3- Summary review. All comments on the text are collated and reviewed by at least two experienced in-house project managers, who also review the text themselves and comment on any additional aspects that may affect the translatability of it, based on their experience with a range of languages. The project managers, who are native English speakers, also assess the language, structure and overall clarity of the source text purely in regard to its usage in English, as there are often opportunities to improve the English source for its own sake, which in turn aids the translatability of it.  4- Consultation with the instrument developer. The project manager creates a report documenting all feedback from the translators and project managers, including proposed recommendations, and provides it to the developer for their review. A teleconference or in-person meeting is then held between the project manager and the developer, in which the comments and recommendations are discussed and the most suitable solution for each item is agreed.  5- Final report and recommendations. The project manager creates a final report detailing all work conducted and presenting the final recommendations for change as agreed with the instrument developer. He also provides an updated version of the source text. |

Analysis of commonalities and disparities/originalities

A minimum of three steps are identified and advised by all: 1) Review of the source text (with substeps); 2 Recommendations; and 3) Report.

Four organizations refer to the identification of concepts as the first step to be performed [i.e., “Identification of concepts” (Mapi and Corporate Translations); “Developers identify intended meaning of concepts” (Health Research Associates); “Concept elaboration” (PharmaQuest)].

Five organizations indicate who the person(s) in charge of each step is(are) (i.e., Corporate Translations, FACITtrans, Health Research Associates, ICON and PharmaQuest).

Three organizations mention the development of a TA table/grid to monitor and perform the review (i.e., Evidera, Health Research Associates and ICON).

Two organizations advise a consultation with the developer before presenting the final recommendations to agree on the most suitable solution for the source text (i.e., Health Research Associates and PharmaQuest).

The major differences are found in the review step:

- In the number of people involved (when specified) [i.e., n=9 (Corporate Translations); n ≥ 3 (FACITtrans); 6 ≤ n ≤ 8 (Health Research Associates); 7 ≤ n ≤ 17 (ICON); 8 ≤ n ≤ 13 (PharmaQuest)];
- In the number and nature of substeps [i.e., three types of reviews (one by a survey research analyst, reviews by professional translators, consolidation and synthesis by survey analyst and project manager - Corporate Translations); review, lexibility assessment, difficulty rating (Evidera); review, synthesis and consolidation (FACITtrans); review, difficulty rating, collation of reviews and combination with on-going cognitive interviews (Health Research Associates); review and compilation (ICON); international and summary reviews (PharmaQuest)].

**S4. People involved**

Table 4 provides an overview of the people involved in the process as suggested by each organization.

**Table 4. People involved**

| **Company / Organization**  **(in alphabetical order)** | **People involved** |
| --- | --- |
| **Corporate Translations, Inc** | - Instrument Developer - Native-speaking translators representing a variety of language groups (typically Western European, Eastern European, Indian, Scandinavian, Asian and Middle Eastern) - Project Manager - Survey Research Analyst |
| **Department of Medical Social Sciences, Northwestern University (NU)** | Translatability assessment is conducted by one experienced reviewer, native speaker of a language other than the source and who has knowledge of other languages; or by a team of reviewers, proficient in a language other than the source, preferably their native language, and experienced in translation.  Instrument developer(s) - to provide clarification about concepts and make final decisions on wording and content of measures. |
| **Evidera, Inc** | - Developer of PRO or COA instrument - Single reviewer with over 18 years of experience in translation and linguistic validation with a range of PRO and COA instruments in over 60 languages |
| **FACITtrans, LLC** | Translatability Assessment should be carried out by professionals with training in the area in conjunction with the item developers. The number of people involved in each assessment can vary according to time and budget. It can be interesting to involve reviewers from different regions (Asia, Europe, etc.). |
| **Health Research Associates, Inc** | COA Development Scientists, Interviewers, Translation Project Managers, Translation consultants |
| **ICON plc** | 2 Senior Project Managers, a number of linguists (depending on client/developer) |
| **Mapi** | - Developer of PRO instrument - Minimum two culturally competent translators - TA coordinator |
| **PharmaQuest Ltd** | - The instrument developer - At least two experienced project managers - Up to 10 translators who are experienced in PRO translation, whose native languages and countries collectively represent as wide a range as possible. |

Analysis of commonalities and disparities/originalities

All companies advise to work with a minimum of two persons. Other than that, the number of people to be involved varies greatly, especially the number of reviewers/translators involved (from 1 to 15). Below is a detailed review of commonalities and discrepancies.

Seven organizations advise to collaborate with the instrument developer (i.e., Corporate Translations, Evidera, FACITtrans, Health Research Associates, Mapi, NU, and PharmaQuest). Of note, the wording used by Health Research Associates seems ambiguous: “COA development scientists” might mean the developer of the measure under review or researchers familiar with or skilled in the development of COAs. ICON mentions the developer, but not clearly as an active part of the team.

To perform the review itself, six organizations recommend to work with a minimum of two people whether they call them “native speaking translators (i.e., Corporate Translations, n=7), reviewers (i.e., FACITtrans, n=variable); translation consultants (i.e., Health Research Associates, n=variable; in the steps category, it is indicated 4 to 6 representative languages), linguists (i.e., ICON, n=variable; in the steps category, 5 to 15 are suggested), culturally competent translators (i.e., Mapi, n≥2), translators experienced in PRO translations (i.e., PharmaQuest, n=up to 10). Only two mention one single reviewer with a robust experience: “18 years, involved in more than 60 linguistic validation projects” (Evidera or “one experienced reviewer, native speaker of a language other than the source and who has knowledge of other language” (NU).

Only two organizations specify the type of languages or the localization of the languages to be spoken by the reviewers. Corporate Translations suggests several language groups, i.e., Western European, Eastern European, Indian, Scandinavian, Asian and Middle Eastern. FACITtrans suggests regions, i.e. Europe, Asia, etc. PharmaQuest recommends that “native languages and countries collectively represent as wide a range as possible.” ICON specifies that the number will depend on the client and the developer. However, a list of languages and a number of people is provided by ICON in the steps category: “review by linguists representing different groups (5-15) (e.g., languages using Chinese characters, Indian languages, Cyrillic languages, Romance and Germanic languages, Latin American Spanish, African languages (Zulu etc.).”

Five organizations advise to work with a minimum of one project manager [i.e., Corporate Translations (n=1); Health Research Associates (n=not specified); ICON (n=2); Mapi (the project manager is called here a “TA coordinator” – n=1); PharmaQuest (n ≥2)]. Regarding their experience, only two companies qualify their project managers (i.e., ICON specifies that they should be “Senior,” and PharmaQuest, that they should be “experienced”).

Besides these three categories of people common to a minimum of five organizations (developer, project manager and translators/reviewers), two companies propose the intervention of a survey research analyst (i.e., Corporate Translations, n=1), or interviewers (i.e., Health Research Associates, n=not specified).

**S5. Timing of Assessment**

Table 5 provides an overview of the timing of assessment proposed by each organization.

**Table 5. Timing of assessment**

| **Company / Organization**  **(in alphabetical order)** | **Timing of assessment** |
| --- | --- |
| **Corporate Translations, Inc** | To be completed during instrument creation, after items are generated and prior to psychometric validation/patient interviews. |
| **Evidera, Inc** | During instrument development, after item generation and construction of the draft instrument but before conducting first round of cognitive interviews. Often in parallel with KOL reviews. Repeated if the instrument is modified during the internal review process.  After each round of cognitive interviews with patients/target population if changes are made to the source wording. |
| **Department of Medical Social Sciences, Northwestern University (NU)** | The translatability review should be performed once the items, instructions or tests are considered almost final, but while wording changes can still be implemented. When cognitive interviews are part of the item/test development methodology, the translatability review should ideally be conducted before the cognitive interviews take place, so that the translatability review input can be incorporated into the interview script. |
| **FACITtrans, LLC** | As early in the process as possible. Ideally, it should be performed during the item development phase while there is still time to make changes to items. Any time a new item is generated it should be reviewed/assessed. |
| **Health Research Associates, Inc** | Process takes 2-4 weeks depending on size of the measure and number of languages involved in the assessment. It is done during the cognitive interview stage where the measure is reasonably well formed but not completely finalized yet from the qualitative stage of development. |
| **ICON plc** | Prior to cognitive debriefing |
| **Mapi** | During instrument development, after item generation and before the first round of cognitive interviews.  Repeated after each iteration of instrument revision during cognitive interviews. |
| **PharmaQuest Ltd** | During the development of the instrument, once a draft format is established, and before large scale validation. The process can be iterative following further testing and revision if required. |

Analysis of commonalities and disparities/originalities

All organizations agree that TA should be conducted during the development phase of the measure. Six organizations clearly specify it [i.e., “during instrument creation” (Corporate Translations); “During instrument development” (Evidera); “during the item development phase” (FACITtrans); “when the measure is reasonably well formed but not completed finalized” (Health Research Associates); “During instrument development” (Mapi); “During the development of the instrument” (PharmaQuest)]. It is assumed that ICON provides the same advice, while stating the following: “prior to cognitive debriefing.”

Then, it is specified at which step of instrument development TA should be conducted:

- Seven organizations state that it should be after item generation [i.e., “after items are generated” (Corporate Translations); “after item generation” (Evidera and Mapi); “once the items, instructions or tests are considered almost final, but while wording changes can still be implemented” (NU); “when the measure is reasonably well formed but not completely finalized” (Health Research Associates); “once a draft format is established” (PharmaQuest)]. It is assumed that ICON provides the same advice, while stating the following: “prior to cognitive debriefing.” FACITtrans specifies “during the item development phase while there is still time to make changes.”
- Five organizations indicate that it should be done prior to patient testing [i.e., “prior to patient interviews” (Corporate Translations); “before conducting first round of cognitive interviews” (Evidera); “Prior to cognitive debriefing” (ICON); “before the first round of cognitive interviews” (Mapi); “before the cognitive interviews” (NU)]. Only Health Research Associates indicate that “it is done during the cognitive interview stage.”
- Two specify that TA should be conducting before the evaluation of the psychometric properties of the measure [i.e., “prior to psychometric evaluation” (Corporate Translations); “before large scale validation” (PharmaQuest)].
- Four organizations advise that the process should be iterative [i.e., “Repeated if the instrument is modified during the internal review process” (Evidera); “Any time a new item is generated it should be reviewed/assessed” (FACITtrans); “Repeated after each iteration of instrument revision during cognitive interviews” (Mapi); “The process can be iterative following further testing and revision if required.” (PharmaQuest)].

The terminology used to qualify patients’ interviews during the qualitative phase of item generation differ [i.e., “patient interviews” (Corporate Translations); “cognitive interviews” (Evidera, Health Research Associates, Mapi, and NU); “cognitive debriefing” (ICON)]. Of note, in the FDA PRO guidance [7] the terms “patient cognitive interviewing” or “patient interviews” are used.

Health Research Associates is the only one to provide an indication of how long TA will take (2-4 weeks).

**S6. Review criteria**

Table 6 illustrates all review criteria used by each organization.

**Table 6. Review criteria**

| **Company / Organization**  **(in alphabetical order)** | **Review criteria** |
| --- | --- |
| **Corporate Translations, Inc** | Process seeks to identify:   - Any concepts that would be difficult to translate for either conceptual or terminology-based reasons; - Any phrases or terms that would be difficult to translate for either conceptual or terminology-based reasons; - Any items or concepts that appear to be culturally specific and therefore inapplicable or confusing to a global audience. |
| **Department of Medical Social Sciences, Northwestern University (NU)** | Assess each item and measure to identify possible linguistic and conceptual difficulties, anticipate translation and relevance issues, consider whether the use of specific technology impacts translation into other languages, and suggest alternative wording solutions more suitable for a culturally diverse population, for translation, and for the instrument’s mode of administration. Classification of issues include: ambiguous, multidimensional concept, idiomatic or jargon, cultural relevance, high register, confusing syntax, etc. |
| **Evidera, Inc** | - Linguistic issues: issues related to semantics, syntax, pragmatics - Cross-cultural issues: issues related to the relevance and suitability of the content in other cultures - Structural issues: issues related to the structure of the item/questionnaire - Reading level issues: if the reading level of the item is too high, simplification suggested whenever possible. |
| **FACITtrans, LLC** | Linguistic issues are evaluated on an item by item basis and can be categorized based on:   - Ambiguity - Cultural appropriateness - Register (too high/too low/different context) - Sentence structure, etc. |
| **Health Research Associates, Inc** | Difficulty level in finding an appropriate and suitable rendering to relay the intended concept (the more difficult, the greater the need for the item to be changed during final development stages) |
| **ICON plc** | None specified up front |
| **Mapi** | - Cross-cultural issues due to differences between source and target languages in terms of culture, semantics, syntax, pragmatics and script/format; - Structural issues, i.e., issues intrinsic to the item/questionnaire structure. |
| **PharmaQuest Ltd** | - All aspects of whether a direct translation (e.g. a translation without any significant semantic or structural changes) would be linguistically possible and appropriate in a range of target languages. - All aspects of whether a direct translation would be culturally appropriate for a range of target countries. - The clarity, language and structure of the source text as an English measure. |

Analysis of commonalities and disparities/originalities

All organizations, except ICON, provide a list of review criteria. Health Research Associates only refers to “difficulty level in finding an appropriate and suitable rendering to relay the intended concept (the more difficult, the greater the need for the item to be changed…).”

The terminology used to qualify the problems identified during the review of the translatability of the source text vary. Four organizations refer to “issues” (i.e., Evidera, FACITtrans, Mapi, and NU), two, to “difficulty” or difficult” (i.e., Corporate Translations and Health Research Associates), and PharmaQuest to the appropriateness of “aspects.”

The cultural specificity of the source/target text/country is mentioned by five organizations (i.e., Corporate Translations, Evidera, FACITtrans, Mapi and PharmaQuest), as well as references to linguistics.

Three organizations refer to the structure of the source text [i.e., “Structural issues” (Evidera and Mapi); “The clarity, language and structure of the source text as an English measure” (PharmaQuest)]. However, it is not clear if they all refer to the same structural issues. PharmaQuest clearly refer to the structure of the source text as an English measure. Evidera and Mapi do not specify what they mean by structure. In the paper published by Conway et al,[10] it seems that “structural issues” refer to the occurrence of double negative in the source text, lack of coherence of text with concept, etc.

Only two organizations share a common view and use the same terminology (i.e., semantics, syntax, pragmatics, cross-cultural and structural) to qualify the criteria to be applied (i.e., Evidera and Mapi), with Evidera providing an original criterion, i.e., the reading level needed to understand an item. Of note, FACITtrans and NU also consider a similar concept but use a different terminology, i.e., “register.”

Only two refer to the difficulty in translating the concepts in the source text [i.e., “Any concepts that would be difficult to translate” (Corporate Translations); “Difficulty level in finding an appropriate and suitable rendering to relay the intended concept” (Health Research Associates)].

PharmaQuest is the only one to mention the possibility or the impossibility to perform a “direct translation.”

The review highlights some sort of confusion between linguistics and culture [i.e., “Issues are evaluated on an item by item basis and can be categorized based on cultural appropriateness” (FACITtrans); “Cross-cultural issues due to differences […] in terms of […] semantics, syntax, pragmatics, script/format” (Mapi)]. If not a confusion, it reveals a vagueness to be corrected.

**S7. Recommendations**

Table 7 lists the recommendations (from all organizations) resulting from the TA process.

**Table 7. Recommendations**

| **Company / Organization**  **(in alphabetical order)** | **Recommendations** |
| --- | --- |
| **Corporate Translations, Inc** | - No changes necessary to existing item; - Recommend specific changes to existing item to ensure adequate translatability; - Recommend that item be dropped. |
| **Department of Medical Social Sciences, Northwestern University (NU)** | -No apparent translatability issue. No change.  -Other languages will phrase it differently but no need to change source.  -Revision of source is recommended, to improve clarity of source and facilitate translations.  -Revision or deletion of source is needed because it’s not translatable as is.  All qualitative information provided by the reviewer(s) is taken into consideration by the instrument developer(s) when making final decisions on the wording and cross-cultural applicability of each measure. |
| **Evidera, Inc** | - No change to the original item wording which can be used as is for translation - No change to the original item wording, but suggestions for alternative wording suitable for translation to address known issues. Suggested when the source wording is the best way to express the concept but doesn’t translate well. - Change to the original item wording, which should be used as the basis for translations - Consider removing the item because of extreme degree of difficulty to translate in the future or because of redundancy between items and the lack of sufficient equivalents in other languages to cover all options |
| **FACITtrans, LLC** | - Change to the source wording necessary. - Change to the source wording not necessary but range of acceptable alternatives for target languages should be decided upon with the developer. - No change to the source wording necessary. |
| **Health Research Associates, Inc** | Levels of difficulty assessed to decide the need to make changes to items  Changes to items recommended in the review process and confirmed in subsequent cognitive interviews |
| **ICON plc** | Suggested changes to wording, sometimes the wording is OK but needs to be well defined in the concept elaboration. |
| **Mapi** | - No change to the original item wording which can be used as such for translation purposes; - No change to the original wording, but translation will have to be based on an alternative formulation; - Change wording of original item; - Original item should be dropped. |
| **PharmaQuest Ltd** | We would either confirm the wording of each item is suitable for international translation and does not require any changes, or we would propose new wording, or we would propose that an item be removed. In some cases we would also agree alternative translation solutions for certain items. In all cases we would recommend that the final concept elaboration be retained and used alongside all future translations. |

Analysis of commonalities and disparities/originalities

The recommendations go from the most elaborated (i.e., Evidera, Mapi, and PharmaQuest) to the simplest (i.e., Health Research Associates and ICON).

All suggest the possibility of not changing the wording of the original or changing it.

The recommendations go from:

- No change to the original (all).

ICON does not clearly says “no change”, but mentions that the fact that “sometimes the wording is OK but needs to be well-defined in the concept elaboration.” Therefore, we have assumed that “no change” is suggested. We have also considered that Health Research Associates suggests the possibility of no change by stating “Levels of difficulty assessed to decide to make changes to items.”

- No change to the original item wording, but suggestions for alternative wording suitable for translation (i.e., Evidera, FACItrans, Mapi, and PharmaQuest).
- Change to the original necessary (all)
- Deletion of original item (i.e., Corporate Translations, Evidera, Mapi, NU, and PharmaQuest).

Evidera is the only organization providing some sort of rationale for each recommendation.

The terminology used to qualify the document under review varies. Most of the organization uses “item” [i.e., Corporate Translations, Evidera, Health Research Associates, Mapi and PharmaQuest). Of note, three of them combine “item” and “wording” (i.e., Evidera, Mapi and PharmaQuest). NU refers to “source” and FACITtrans combines “source” and “wording.” ICON only uses the term “wording.” Our analysis reveals that there is a lack of precision in the use of terminology to qualify the document under review. It is not clearly indicated that all the elements of the PRO measure (i.e., instructions, items, and response categories) should be reviewed and that decisions should be made for each element.

Only one organization suggests that the final concept elaboration be retained for future translations (i.e., PharmaQuest).

Only NU seems to mention that the recommendations are not prescriptive and it is up to the developer(s) of the original measure to agree upon them [i.e., “All qualitative information provided by the reviewer(s) is taken into consideration by the instrument developer(s) when making final decisions on the wording and cross-cultural applicability of each measure.”]
